# Nuclear Magnetic Resonance (NMR) Study for the Detection and Quantitation of Cholesterol in HSV529 Therapeutic Vaccine Candidate

**DOI:** 10.1016/j.csbj.2016.10.007

**Published:** 2016-11-01

**Authors:** Rahima Khatun, Howard Hunter, Webster Magcalas, Yi Sheng, Bruce Carpick, Marina Kirkitadze

**Affiliations:** a Department of Biology, York University, 4700 Keele Street, Toronto, Ontario, Canada; b Analytical Research & Development, Sanofi Pasteur Ltd., 1755 Steeles Avenue West, Toronto, Ontario, Canada

**Keywords:** Herpes simplex virus type 2 (HSV-2), Viral vaccine, NMR, Residuals, LOD and LOQ, TLC, Growth supplement

## Abstract

This study describes the NMR-based method to determine the limit of quantitation (LOQ) and limit of detection (LOD) of cholesterol, a process-related impurity in the replication-deficient Herpes Simplex Virus (HSV) type 2 candidate vaccine HSV529. Three signature peaks from the 1D ^1^H NMR of a cholesterol reference spectrum were selected for the identification of cholesterol. The LOQ for a cholesterol working standard was found to be 1 μg/mL, and the LOD was found to be 0.1 μg/mL. The identity of cholesterol, separated from the formulation of growth supplement by thin layer chromatography (TLC), was confirmed by 1D ^1^H NMR and 2D ^1^H-^13^C HSQC NMR. The three signature peaks of cholesterol were detected only in a six-times concentrated sample of HSV529 candidate vaccine sample and not in the single dose HSV529 vaccine sample under similar experimental conditions. Taken together, the results demonstrated that NMR is a direct method that can successfully identify and quantify cholesterol in viral vaccine samples, such as HSV529, and as well as in the growth supplement used during the upstream stages of HSV529 manufacturing.

## 1 Introduction

HSV529 is a replication deficient viral vaccine candidate (*d15–29*) against genital herpes caused by the Herpes Simplex Virus type-2 strain. It is engineered by deleting two early protein genes – UL5 and UL29 – which renders it incapable of viral DNA replication [1]. To date, HSV529 has been proven safe, effective and shows promising immunogenicity in animal models [1]. Currently, this viral vaccine candidate is being tested in a clinical in Phase I study [2], [3], [4].

Vaccine development, like any drug development, is a long and rigorous process to ensure the safety of the end user. Multiple testing, such as monitoring process-related impurities, is required to ensure the integrity of the final product.

To assess impurities, a list of all materials used in the manufacturing of HSV529 was compiled along with their estimated concentrations in the final HSV529 Drug Product. These estimations were calculated using the “worst case” scenarios, e.g. by assuming no removal of components during the processing. The growth supplement, as one of the components used, is an additive required for the growth of the cells used in the upstream fermentation. It was estimated to be present at a concentration of 5.3 μg/dose, where each dose is 0.5 mL.

Cholesterol is one of the components in the growth supplement. Although there is no toxicological assessment available for cholesterol, the acceptable maximum cholesterol level set for this vaccine candidate is 3 μg/mL (1.5 μg/dose). This limit was calculated using the FDA Guidance for Industry “Genotoxic and Carcinogenic Impurities in Drug Substances and Products: Recommended Approaches” (2008) [5] and the EMA/CHMP/QWP/251,344/2006 guideline [6]. Both guidelines consider an acceptable maximum of 1.5 μg/dose for genotoxic impurities lacking toxicological assessments. While there is no indication that cholesterol is genotoxic or carcinogenic, this level is used as an initial target for the purpose of defining desirable limits of detection and of quantification.

Plausible analytical methods to assess the cholesterol content include gas–liquid chromatography or liquid chromatography, coupled with flame ionization or mass spectrometry detection [7], [8], [9], and fluorometric [12] or colorimetric enzymatic assays [10], [11]. The chromatography and mass spectrometry methods are relatively time consuming and require chemical saponification of cholesterol esters. The colorimetric and fluorometric enzymatic methods, on the other hand, are simple, sensitive, and relatively faster assays that can measure the total cholesterol content, and cholesterol mass in cholesterol esters [13]. However, the aim was to determine the residual cholesterol content remaining from the growth supplement without further modification, i.e. without the esterification of cholesterol. Therefore, Nuclear Magnetic Resonance (NMR) spectroscopy was chosen as the method of choice as it can analyze the lyophilized vaccine reconstituted in chloroform without further sample treatment.

NMR is routinely used in academia for the study of biomolecules. This method has been proven to be an important tool to characterize newly synthesized peptides, polymers, natural compounds and metabolites [14], [15], [16]. Recent studies demonstrated that NMR can be used routinely for the reliable quantification of small molecules to assess the product's purity [14], [20], [21], [23], [24]. It has been used to identify and quantify polysaccharides in protein conjugate vaccines such as Hib and meningococcal capsular polysaccharides [17], [18], [19]. NMR utilizes various techniques based on the particular nucleus under study, e.g., proton-proton, proton-carbon, and proton-nitrogen. Quantitative information is extracted from the signal integrals – resonance lines that do not overlap with other resonances – that are linearly proportional to the concentration of the analyte. Conventionally, a reference substance of known concentration is run along the sample to calculate the concentration of the different constituents [14].

Previously, an attempt was made to detect cholesterol in aqueous solutions of the HSV529 candidate vaccine and the growth supplement using NMR; however, it was not successful (data not shown). To further assess the NMR method in evaluating cholesterol levels, cholesterol was extracted from the HSV529 candidate vaccine and growth supplement using chloroform prior NMR analysis.

Since there are no internal standards for cholesterol, the Limit of Quantification (LOQ) and Limit of Detection (LOD) of cholesterol were first determined using a reference standard (Caledon Laboratories Ltd). These limits were then used to assess the presence of cholesterol in the growth supplement and in the HSV529 candidate vaccine. In summary, the objectives of this study were to develop NMR methods to: (a) identify and quantify cholesterol as a process-related impurity in HSV529, and (b) assess the presence of cholesterol in the growth supplement. The samples analyzed in this study include the cholesterol standard, the growth supplement and the HSV529 vaccine candidate.

## 2 Materials and Methods

### 2.1 Key Data Acquisition Parameters

A Bruker AVANCE 700 MHz Nuclear Magnetic Resonance Spectrometer equipped with a 5 mm cryoprobe and a Z-axis pulse field gradient was used throughout this study. The key acquisition parameters for 1D ^1^H NMR protein antigen sample spectra were as follows: eight and 512 scans, a relaxation delay of five seconds, 90° pulsewidth of 8 μsec at a transmitter power of 7.59 W, temperature of 25 °C, acquisition time of 2.32 s, and spectral width (swh) 14,097.7 Hz with 64 K acquired data points. The FID signal was locked using the CDCl_3_ solvent and then optimized for magnetic field homogeneity (shimmed). The 90° pulsewidth was calibrated prior to the experiment.

### 2.2 LOQ/LOD for Cholesterol Powder

To determine the optimum scan number, cholesterol powder (Caledon Laboratory Chemicals) was dissolved in CDCl_3_ (99.8%, Sigma-Aldrich) and loaded into an NMR tube in concentrations of 10, 1 and 2500 μg/mL, respectively. 1D ^1^H NMR spectra were collected at 8 versus 512 scans in 300 μl volume using 3 mm NMR tube as described in Section 2.1. For LOQ and LOD, 1D ^1^H NMR spectra were obtained for a series of cholesterol dilutions in CDCl_3_ at 100, 10, 1 and 0.1 μg/mL, respectively. For each dilution, 512 scans were collected in the same experimental conditions. Both experiments were repeated using 5 mm NMR tube and the spectra were processed using NMR software TOPSPIN version 3.1 and compared with the 1D ^1^H NMR spectrum of cholesterol in CDCl_3_ [22].

### 2.3 Detection of Cholesterol in Growth Supplement

According to the supplier's product description, the growth supplement is an aqueous cholesterol solution in a proprietary formulation. As shown in Supplement 1 (Fig. S1), previous 1D ^1^H NMR experiments suggested the presence of unknown components in the growth supplement solution that overlapped with cholesterol's signals [2]. In an attempt to separate cholesterol from the other components in the growth supplement solution, a thin layer chromatography (TLC) method [21] was employed prior to 1D ^1^H NMR measurement.

TLC is a separation technique for non-volatile compounds, and is performed on a sheet of glass, plastic, or aluminum foil that is coated with a thin layer of adsorbent material. In this study, a TLC plate was used with silica gel as stationary phase. 5 μl of growth supplement and reference cholesterol samples (2500 μg/mL in CDCl_3,_ Caledon Laboratory Chemicals) were loaded into two different slots at the bottom of the plate (Fig. 3). The plate was then eluted at room temperature using a benzene-d_6_:ethyl acetate:glacial acetic acid (Sigma Aldrich) mixture in a 60:40:1 ratio. After elution, the plate was covered with a ceric ammonium molybdate solution (Sigma Aldrich) and heated. The ceric ammonium nitrate (Sigma Aldrich) was only used on a test plate to measure the retention index. This retention index was measured on a preparative plate and the region containing cholesterol was collected (see Fig. 3) and dissolved in 300 μl CDCl_3_ to make the NMR sample. Therefore, ceric ammonium nitrate did not come in contact with the cholesterol used for NMR analysis. 1D ^1^H and 2D ^1^H-^13^C NMR data (512 scans) were collected as described in Section 2.1 and spectra were processed using TOPSPIN software version 3.1.

### 2.4 Detection and Quantitation of Cholesterol in HSV529

To detect residual cholesterol in the candidate vaccine, two separate cholesterol extractions were performed. Since cholesterol is insoluble in water (critical micellar concentration of 1.3–6.5 X 10^− 8^ M [25]), chloroform solvent extraction method was chosen to isolate cholesterol. In the first extraction experiment, 0.5 mL of 99.8% CDCl_3_ was added to each vial (0.5 mL) of lyophilized HSV529 therapeutic vaccine. Samples were sonicated for 10 min in a water bath and the clear chloroform solution was collected into two samples: (a) single dose sample and (b) 6X concentrated sample. CDCl_3_ was evaporated using a rotary evaporator and the pooled sample was re-dissolved in 500 μl of 99.8% CDCl_3_ prior collecting the 1D ^1^H NMR. Data were collected in three replicates and 2D ^1^H-^13^C NMR and cholesterol spiking were performed for six-times concentrated sample only as the 1D ^1^H NMR data did not identify cholesterol in a single dose (0.5 mL sample). Finally, the spectra were processed and analyzed.

The amount of cholesterol present in two of the three aliquots from the previous section was determined by spiking with the standard cholesterol. Using a 10 μg/mL solution of cholesterol in CDCl_3_, a 50 μL spike was added to each of two aliquots and the spectra were recorded using identical conditions and parameters used in the previous experiment. After processing the spectra similarly to the original spectra (i.e. 1 zero fill, 0.3 Hz line broadening and standard polynomial baseline correction), the original and the spiked spectra were scaled relative to each other and compared to calculate the difference in the peak heights. The cholesterol concentrations were determined using the following relationship:

$\frac{\mathrm{Cx}}{\mathrm{Cx}+\mathrm{Cs}}=\frac{\mathrm{Signal}\left(x\right)}{\mathrm{Signal}\left(x+s\right)},$

where:

Cx: unknown concentration.
Cs: concentration of the standard in the spiked solution (i.e. 10 μg/mL x (50 μL ÷ 550 μL) = 0.91 μg/mL).
Signal(x): original resonance intensity (set to 1 in each case).
Signal(x + s): resonance intensity after addition of the cholesterol spike.

## 3 Results and Discussion

### 3.1 LOQ of Cholesterol Is 1 μg/mL and LOD of Cholesterol Is 0.1 μg/mL

Previously, assessment of the presence of residual growth supplement or the cholesterol for the HSV529 vaccine candidate was performed in aqueous solutions. The 1D ^1^H NMR spectra of the growth supplement and the HSV529 vaccine samples showed no trace of cholesterol in either sample. The 1D ^1^H NMR spectrum of the growth supplement showed no characteristic signature peaks of cholesterol. Furthermore, HSV529 vaccine candidate in aqueous solution spectrum showed no presence of the growth supplement.

These observations led to changes in the sample preparation methods for the growth supplement and the HSV529 vaccine, which in turn allowed the measurement of residual cholesterol at the microgram level by NMR. The LOQ and LOD of cholesterol by NMR were determined first.

**Fig. 1:**
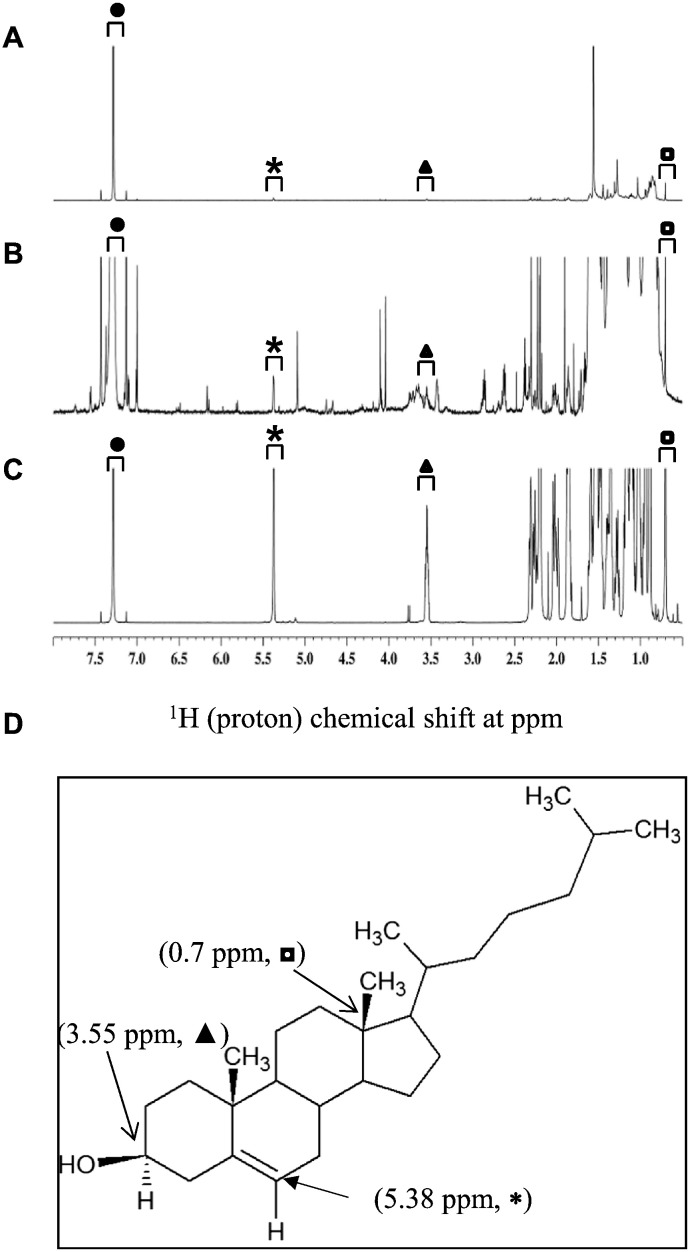
Determination of scan numbers required for LOQ and LOD of cholesterol in CDCl_3_ using 1D ^1^H NMR. The spectra show the ^1^H chemical shifts of cholesterol at 10 μg/mL (panel A, 8 scans) 1 μg/mL (panel B, 512 scans) and 2500 μg/mL (panel C, 8 scans). Three non-overlapping, signature peaks are indicated as asterisk (*), triangle (▲) and rectangle (◘) at 5.38, 3.55 and 0.7 ppm, respectively, that have unique chemical shifts for cholesterol. Closed circle (●) indicates the peak for chloroform at 7.5–7.0 ppm. Panel D shows the cholesterol structure with the unique protons identified.

The 1D ^1^H NMR spectrum of cholesterol indicates many aliphatic proton resonances in the chemical shift ranges of 2.4–0.6 ppm, as well as unique resonances at 5.38, 3.55 and 0.70 ppm (Fig. 1 panels A-C). The unique resonances at 5.38, 3.55 and 0.70 ppm were assigned to cholesterol, since there was very little, if any, spectral overlap from other compounds in these chemical shift regions [22], [26]. To determine the optimal number of scans required for quantitation of cholesterol, data were collected at 8 versus 512 scans at different concentrations. As shown in Fig. 1 (panel A), the 8-scan spectra of cholesterol showed signature peaks at 5.38, 3.55 and 0.70 ppm (chemical shift and peak area) for the 10 μg/mL of cholesterol. Furthermore, using 512 scans, signature peaks were detected at the concentration as low as 0.1 μg/mL of cholesterol indicating that the signature peaks of cholesterol are detectable at microgram level as shown in Fig. 1 panel B. Fig. 1 panel C shows that a higher cholesterol concentration (2500 μg/mL, 8 scans) was used as a positive control.

**Fig. 2:**
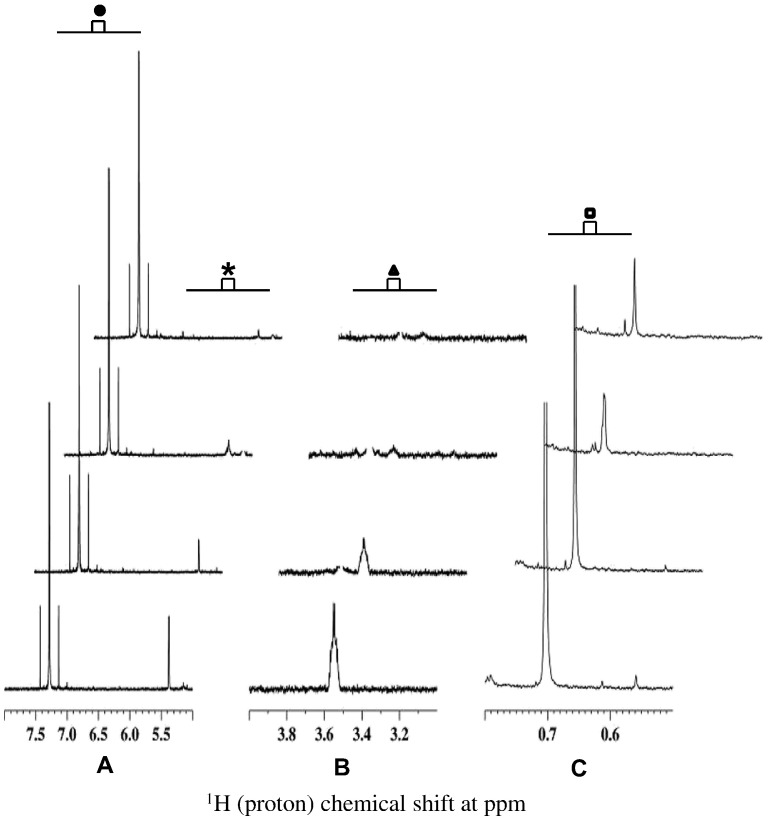
1D ^1^H NMR spectra of cholesterol in CDCl_3_ at four different dilutions. Spectra are stacked from top to bottom at the following order: 0.1 (top), 1 (2nd top), 10 (3rd top) and 100 (bottom) μg/mL, respectively. 512 scans for each dilution are used and shown as offset to the right of the axis (bottom to top). Three non-overlapping signature peaks for cholesterol are indicated as asterisk (*, panel A), triangle (▲, panel B) and rectangle (◘, panel C) at 5.38, 3.55 and 0.7 ppm, respectively. These peaks have unique chemical shifts for cholesterol. Closed circle (●) indicates the peak for chloroform at 7.5–7.0 ppm that is used as internal reference to show the scale.

Next, to determine the LOQ and LOD, a series of dilutions of cholesterol by factor of 10 were analyzed by 1D ^1^H NMR. As shown in Fig. 2, cholesterol signals were decreased as a result of dilution from 100 μg/mL to 1 μg/mL. All three signature peaks were still visible and quantifiable at 1 μg/mL; therefore, the LOQ of cholesterol was found to be 1 μg/mL. At the concentration of 0.1 μg/mL only two signature peaks of 5.38 and 0.7 ppm were clearly visible; whereas, the peak at 3.55 ppm is missing. Therefore, LOD is claimed to be 0.1 μg/mL. Although it would be preferable to have all three signature peaks for LOD, from safety point of view, it is important to consider the HSV529 sample where at least one peak of cholesterol is detectable.

### 3.2 1D/2D NMR Is Useful to Detect Cholesterol in Growth Supplement Sample at LOD Level

**Fig. 3:**
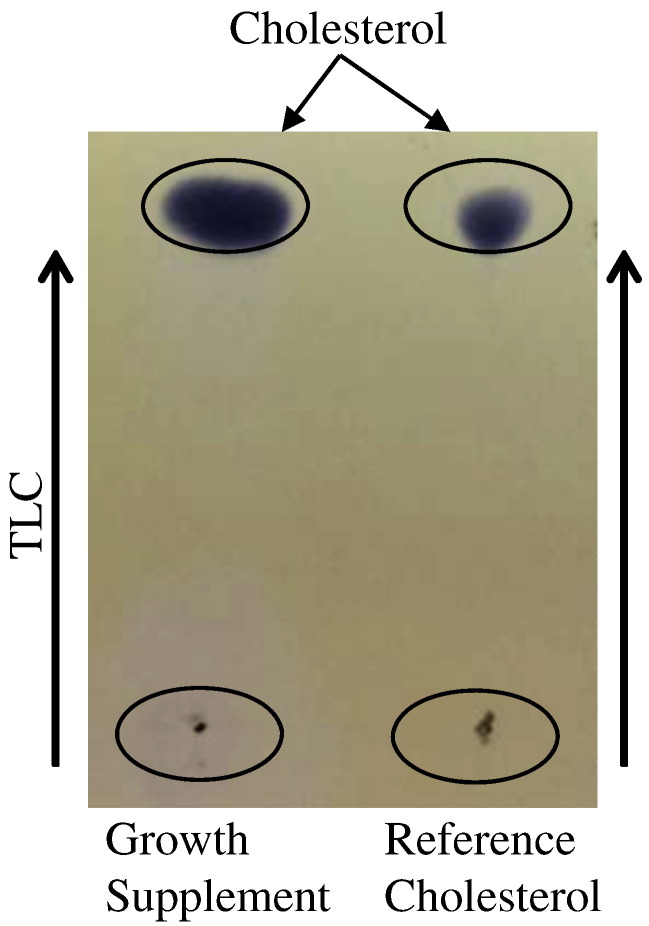
TLC extraction of cholesterol from growth supplement. 5 μL of each sample was inserted into the corresponding slots at the bottom of the plate as indicated. After development, cholesterol for growth supplement were scrapped off and dissolved in CDCl_3_ for NMR experiment.

**Fig. 4:**
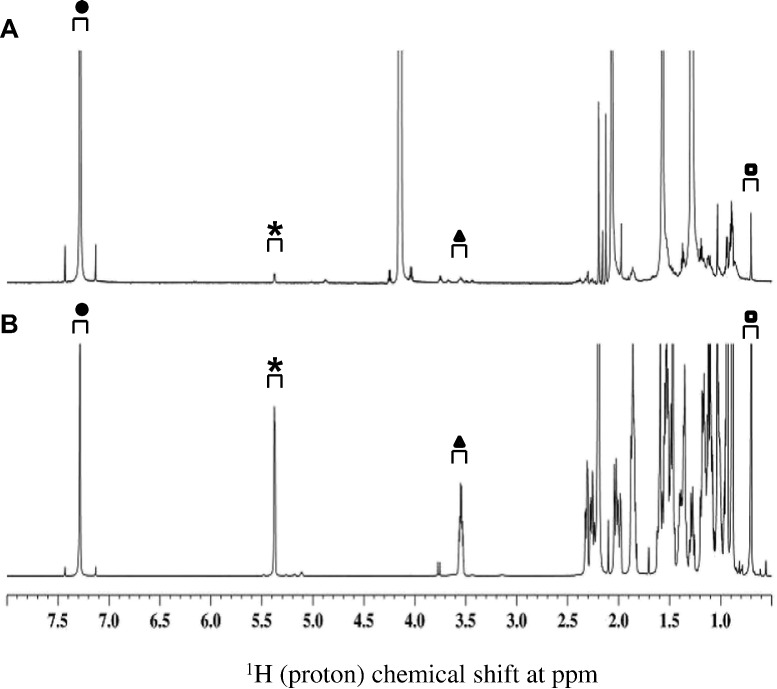
1D ^1^H NMR identified cholesterol in growth supplement sample after preparative TLC isolation. Panel A represents the spectrum of cholesterol isolated from growth supplement, panel B represents the reference cholesterol spectrum. Three non-overlapping, signature peaks are indicated as asterisk (*), triangle (▲) and rectangle (◘) at 5.38, 3.55 and 0.7 ppm, respectively, that have unique chemical shifts for cholesterol. Closed circle (●) indicates the peak for chloroform at 7.5–7.0 ppm.

**Fig. 5:**
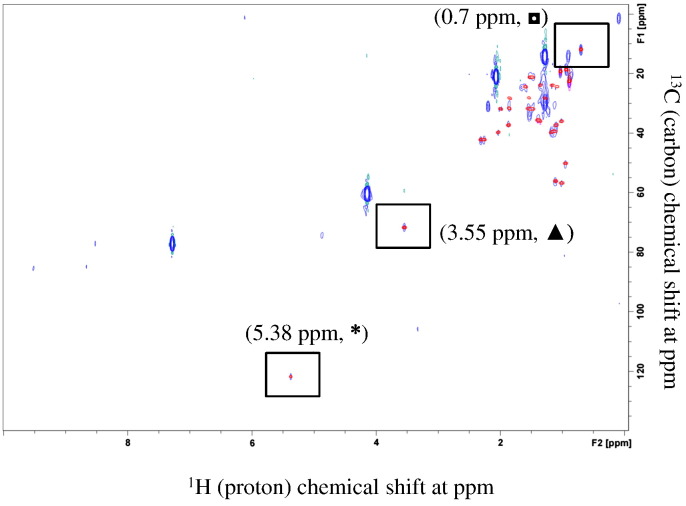
2D ^1^H-^13^C NMR detected cholesterol signature peaks in TLC isolated cholesterol from growth supplement sample. 2D ^1^H-^13^C NMR spectra for cholesterol of growth supplement (blue spots) and reference cholesterol (red spots) are aligned. Superimposed three signature peaks at 5.38, 3.55 and 0.7 ppm are shown within square boxes where ^1^H-^13^C cross peaks were identified for both samples. (For interpretation of the references to color in this figure legend, the reader is referred to the web version of this article.)

Previous 1D ^1^H NMR experiments suggested the presence of additional unknown components in the formulation of the growth supplement with signals overlapping with the cholesterol signals [22]. To examine growth supplement, cholesterol was isolated from the proprietary formulation using TLC. As shown in Fig. 3, the retention time for the spot from the growth supplement was the same as cholesterol standard, indicating that cholesterol was separated from growth supplement. Fig. 4 shows the 1D ^1^H NMR showed cholesterol spectrum with three signature peaks. Furthermore, 2D ^1^H-^13^C HSQC confirmed the presence of cholesterol in the growth supplement. (See Fig. 5.)

**Fig. 6:**
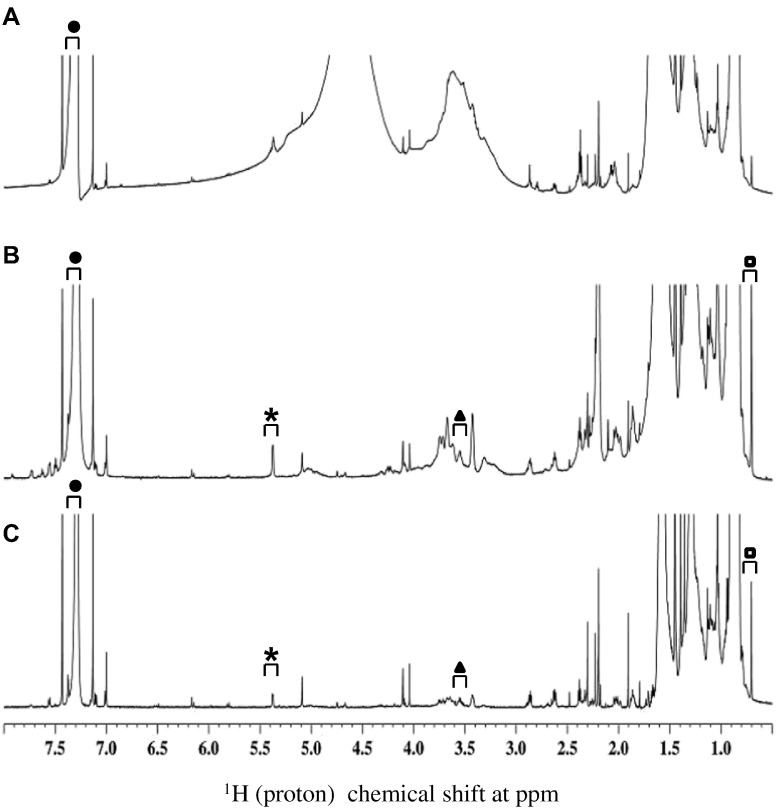
1D ^1^H NMR detected cholesterol in concentrated HSV529 vaccine sample. Panel A and B represent spectrum for 0.5 mL (single dose), and 6 times concentrated HSV529 vaccine samples, respectively. Panel C shows the LOQ spectrum of reference cholesterol (1 μg/mL of cholesterol). Panel A shows the representative spectrum for 0.5 mL and Panel B is the representative spectrum for 6 times concentrated HSV529 vaccine. Three non-overlapping, signature peaks are shown as asterisk (*), triangle (▲) and rectangle (◘) at 5.38, 3.55 and 0.7 ppm, respectively, that have unique chemical shifts for cholesterol. Closed circle (●) indicates the peak for satellite cholesterol from chloroform at 7.5–7.0 ppm.

**Fig. 7:**
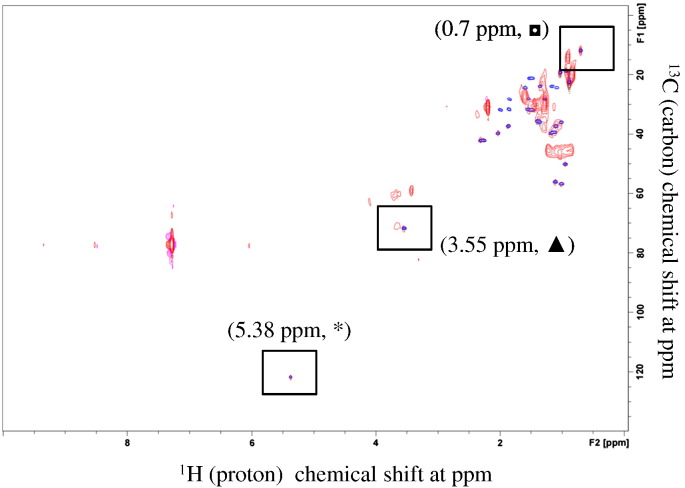
2D ^1^H ^13^C NMR detected cholesterol signature peaks in 6 times concentrated HSV529 sample: 2D ^1^H-^13^C NMR spectra for cholesterol of HSV529 (red spots) and reference cholesterol (blue spots) are aligned. Superimposed three signature peaks at 5.38, 3.55 and 0.7 ppm are shown within square boxes where ^1^H-^13^C cross peaks were identified for both samples. (For interpretation of the references to color in this figure legend, the reader is referred to the web version of this article.)

### 3.3 1D/2D NMR can Quantify Cholesterol in Concentrated HSV529 Vaccine Sample in Microgram Level

Our previous experiments did not detect cholesterol in HSV529 vaccine sample in aqueous solution, possibly due to the fact that: (a) cholesterol is insoluble in water (critical micellar concentration of 1.3–6.5 X 10–8 M [25]), and (b) larger vaccine components mask the smaller cholesterol molecule. Therefore, to detect and quantitate the residual cholesterol in HSV529 vaccine, cholesterol was extracted in chloroform and examined by 1D/2D NMR. As shown in Fig. 6 panel A, for the single dose of HSV529, the baseline of the spectrum became far wider and none of the three signature peaks were identified. In contrast, as shown in Fig. 6 panel B all three signature peaks were identified based on LOQ of reference cholesterol. Furthermore, cross-peaks between proton-carbon for the three peaks were confirmed using 2D ^1^H-^13^C HSQC as shown in Fig. 7. Table 1 shows the cholesterol concentration in two concentrated vaccine samples through spiking with reference cholesterol as described and in Section 2.4. The amount of cholesterol were found to be in the range of 1.08 μg/mL - 1.92 μg/mL, calculated using the formula described in Section 2.4 which was near to the LOQ of 1 μg/mL of cholesterol standard. Taken together, NMR methodologies employed in this study has proven to be useful to study cholesterol in complex biological molecules, like HSV529 vaccine.

**Table 1:** Determination of cholesterol concentration in concentrated HSV529 samples.

Sample #	Resonance Shift (δ ppm)	Signal(x + s) (relative to the original intensity of 1 unit)	Corrected^1^ Concentration (μg/mL)
1	0.70	1.92	1.08
1	5.37	1.52	1.92
2	0.70	1.74	1.34
2	5.37	1.69	1.44

1. The concentrations of the samples were adjusted to account for a loss of resonance intensity caused by dilution from 500 μl to 550 μl.

## 4 Conclusion and Recommendations

^1^H NMR is a well-established method to identify and characterize small chemicals in the pharmaceutical industry [23], [24]. In this study, NMR spectroscopy was evaluated for the identification and quantification of a process-related impurity in vaccine candidate. The immediate goal was to develop a method to monitor cholesterol levels in HSV529 vaccine samples. The developed 1D ^1^H NMR and 2D ^1^H-^13^C NMR methods were shown to be suitable for the quantification and identification of cholesterol in HSV529 samples.

Initially, the LOD and LOQ were determined using a commercial cholesterol sample, which also served as a standard. The LOQ and the LOD measured by the 1D ^1^H NMR method developed were estimated at 1.0 μg/mL and 0.1 μg/mL, respectively. The LOQ was determined using a series of dilutions of a cholesterol standard. The LOD was determined by further dilutions and based on the three signature peaks of the reference cholesterol spectrum [22].

Based on the LOD/LOQ, cholesterol was detected in HSV529 samples concentrated six times. This result was further confirmed using 2D ^1^H-^13^C NMR methods. The cholesterol concentrations in the concentrated HSV529 samples were varied from 1.08 to 1.92 μg/mL. However, cholesterol levels measured in non-concentrated samples of HSV529 were below the LOD, which is likely due to a masking effect. HSV viral particles, host cell proteins, and most likely a biological stabilizer added during the process can be responsible for matrix effect. To mitigate the matrix effect that is masking cholesterol, HSV529 final filled Drug Product will be analyzed.

The 1D ^1^H NMR and 2D ^1^H-^13^C HSQC spectra confirmed the presence of cholesterol in a fraction of the growth supplement samples extracted using a TLC method. Routine NMR spectrometers without cryoprobe can be also used for the detection and quantitation of cholesterol. However, to generate a comparable spectrum, a longer acquisition time and greater number scans will be required.

In conclusion, the results of this study demonstrated that the NMR method can successfully identify and quantify cholesterol in the HSV529 vaccine candidate and in growth supplement used during the upstream stages of vaccine manufacturing.

The following is the supplementary data related to this article.

Supplement 1 Growth supplement Solution (Sigma Aldrich) and Cholesterol Experimentally Obtained Spectra. Supplement 1

## Conflict of Interest

Bruce Carpick, Webster Magcalas, and Marina Kirkitadze are the employees of Sanofi Pasteur Ltd. Rahima Khatun, Yi Sheng, and Howard Hunter are the employees of York University. The authors have no relevant affiliations or financial involvement with any organization or entity with a financial interest in or financial conflict with the subject matter or materials discussed in the manuscript.

No writing assistance was utilized in the production of this manuscript.

## Executive Summary

**Background**

• This study describes the NMR spectroscopy method to determine the limit of quantitation (LOQ) and limit of detection (LOD) of cholesterol, a process-related impurity in the replication-deficient Herpes Simplex Virus (HSV) type 2, HSV529, candidate vaccine samples.

**Method development study design**

• Three signature peaks from the 1D ^1^H NMR of a cholesterol reference spectrum selected for the identification of cholesterol were used to measure the LOQ and LOD, to identity of cholesterol separated from the formulation of growth supplement, and to detect it in six-times concentrated sample of HSV529 candidate vaccine.

**Results and discussion**

• The LOQ for a cholesterol working standard was found to be 1 μg/mL, and the LOD was found to be 0.1 μg/mL. The identity of cholesterol separated from the formulation of growth supplement by TLC was confirmed by 1D ^1^H NMR and 2D ^1^H-^13^C HSQC NMR. Three signature peaks of cholesterol were detected only in a six-times (3 mL) concentrated sample of HSV529 candidate vaccine sample, however, they were not found in the HSV529 vaccine sample (0.5 mL sample volume).

**Conclusions & recommendations**

• The results demonstrated that NMR is a direct method that can successfully identify and quantify cholesterol in viral vaccine samples, such as HSV529, and as well as in the growth supplement used during the upstream stages of HSV529 manufacturing. The method can be applied to examine the impact of the eventual process scale-up and formulation on cholesterol level in of HSV529 candidate vaccine.
