# Supplementary material for: Nuclear Magnetic Resonance (NMR) Study for the Detection and Quantitation of Cholesterol in HSV529 Therapeutic Vaccine Candidate
Source: Comput Struct Biotechnol J. 2016 Nov 1;15:14–20. doi: 10.1016/j.csbj.2016.10.007 (PMC5484764; doi:10.1016/j.csbj.2016.10.007)
Supplement: Supplement 1 — Growth supplement Solution (Sigma Aldrich) and Cholesterol Experimentally Obtained Spectra. [file mmc1.pptx]

## Slide 1
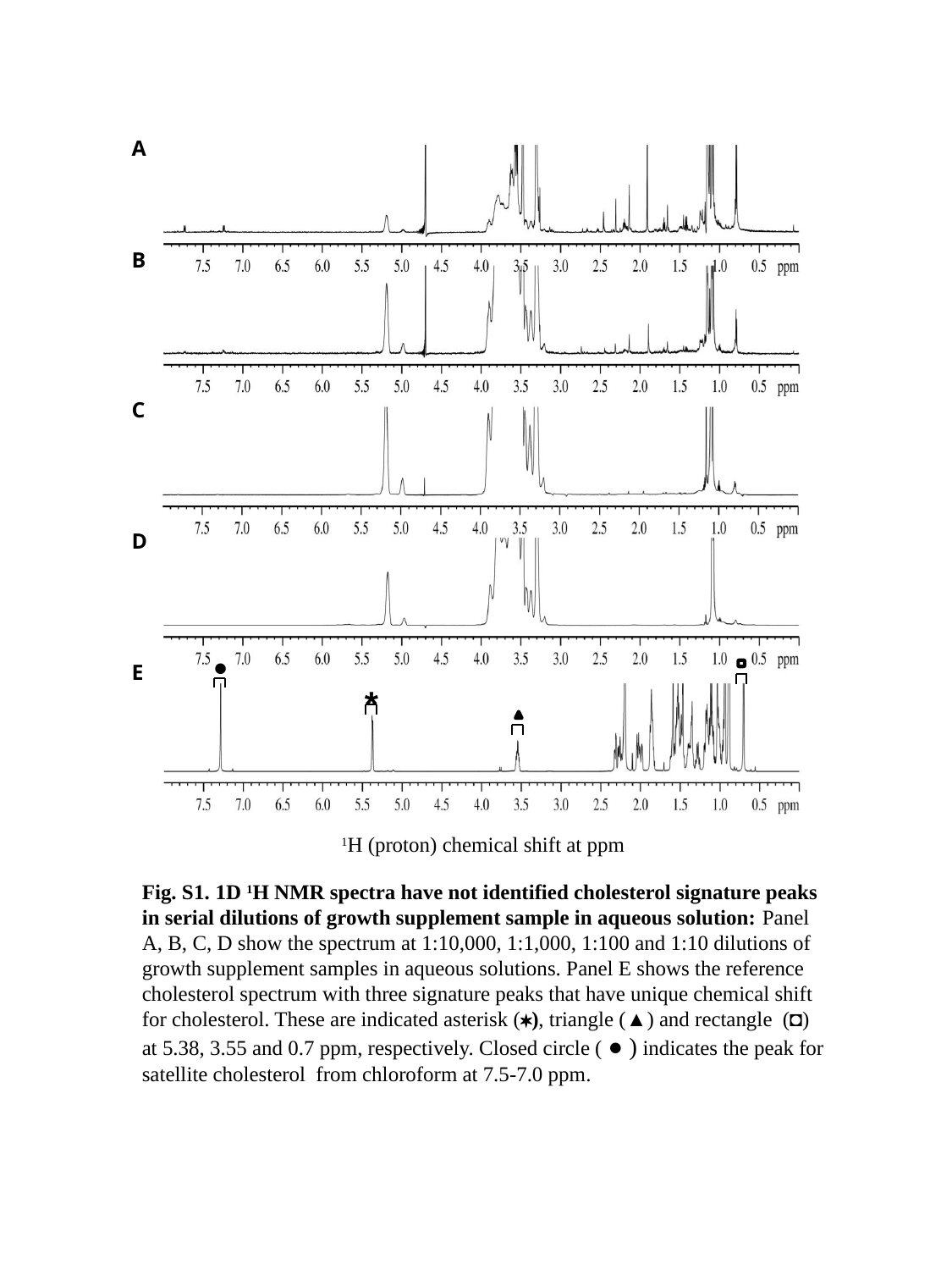

A
B
C
D
E
*
●
1H (proton) chemical shift at ppm
Fig. S1. 1D 1H NMR spectra have not identified cholesterol signature peaks in serial dilutions of growth supplement sample in aqueous solution: Panel A, B, C, D show the spectrum at 1:10,000, 1:1,000, 1:100 and 1:10 dilutions of growth supplement samples in aqueous solutions. Panel E shows the reference cholesterol spectrum with three signature peaks that have unique chemical shift for cholesterol. These are indicated asterisk (), triangle (▲) and rectangle (◘) at 5.38, 3.55 and 0.7 ppm, respectively. Closed circle ( ● ) indicates the peak for satellite cholesterol from chloroform at 7.5-7.0 ppm.
